# Multi-attack and multi-classification intrusion detection for vehicle-mounted networks based on mosaic-coded convolutional neural network

**DOI:** 10.1038/s41598-022-10200-4

**Published:** 2022-04-15

**Authors:** Rong Hu, Zhongying Wu, Yong Xu, Taotao Lai

**Affiliations:** 1 Fujian Provincial Key Laboratory of Big Data Mining and Application, Fujian University of Technology, Fuzhou, China; 2 Fujian Key Laboratory of Automotive Electronics and Electric Drive, Fujian University of Technology, Fuzhou, China; 3 Fujian Provincial Key Laboratory of Information Processing and Intelligent Control, Minjiang University, Fuzhou, China

**Keywords:** Computer science, Information technology

## Abstract

With the development of Internet of vehicles, the information exchange between vehicles and the outside world results in a higher risk of external network attacks to the vehicles. The attack modes to the most widely used vehicle-mounted CAN bus are complex and diverse, but most of the intrusion detection approaches proposed by now can only detect one type of attack at a time. Aiming at detecting multi-types of attacks using a single model, we proposed a detection method based on the Mosaic-coded convolution neural network for intrusions containing various combinations of attacks with multi-classification capability. A Mosaic-like two-dimensional data grid was created from the one-dimensional CAN ID for the CNN to effectively extract the data features and maintain the time connections between the CAN IDs. Four types of attacks and all possible combinations of them were used to train and test our model. The autoencoder was also used to reduce the dimensionality of the data so as to cut down the model’s complexity. Experimental results showed that the proposed method was effective in detecting all types of attack combinations with high and stable multi-classification ability.

## Introduction

With the advancement of information and Internet of things (IoT) technology, intelligent connected vehicles (ICVs) have become a new trend of automobile development. ICVs refer to a new generation of vehicles that are equipped with advanced chips and sensors, connected to the Internet, and possessed with complex environment perception and intelligent control capability^1^. The increase of automobile interfaces and the more and more frequent information exchange between automobile equipment and the outside world lead to the greatly increased risk of the automobile network being attacked by hackers^2^. Network attacks to cars will cause problems such as owner information leakage and vehicle loss of control, which seriously threaten the safe running of cars and roads. Attackers can launch attacks on connected vehicles by means of wired physical access and/or wireless remote access. Since the network security risks faced by automobiles are complex and diverse, and the ultimate goal of network attacks against automobiles is to interfere with the vehicle-mounted network (VMN) of automobiles, the VMN protection of automobiles has become a research hotspot^3^.

**Figure 1:**
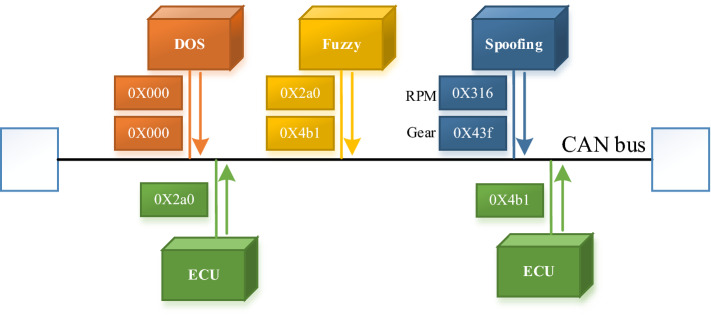
Illustration of the multiple attacks.

The VMN of a car is a kind of network structure to realize the communication inside the car. Controller area network (CAN) is the most common VMN structure and has become the de facto standard of it. However, due to the lack of corresponding security mechanism in the CAN bus network, it is possible for hackers to attack it^4^. Common attack types to the VMNs include (1) Denial of Service (DoS) Attack. As CAN bus transmits messages with priority, in DoS attacks, attackers will continuously send messages with the highest priority to occupy CAN communication resources. As a result, the system cannot send any normal message. (2) Fuzzy Attack. An attacker randomly injects messages into the CAN bus by simulating legitimate messages into the CAN bus, which may cause problems such as steering wheel shaking, signal lights turning on/off at irregular times, and automatic shift. (3) Spoofing Attack. In spoofing attack, the attacker chooses to inject a message with a specific CAN ID into the CAN bus to achieve the purpose of causing vehicle anomalies. Compared to Fuzzy attack, Spoofing attack is to select CAN ID normally transmitted on the CAN bus to attack the network, while Fuzzy attack can create any simulated mock ID to launch the attack. Figure 1 shows a scenario in which multiple of the above attack types are simultaneously attacked on the CAN bus.

The VMN carried various electronic control units (ECUs) to control the vehicles and attackers can invade the VMN by taking control of the ECUs^5^. In recent years, vehicle network attacks occurred frequently, highlighting the seriousness of vehicle network security issues. Miller et al. proved a safety flaw in a Jeep Cherokee by attacking its engine and steering functions, leading to the recall of about 1.4 million vehicles^6^. Checkoway et al. proposed three different attack methods: direct physical attack based on distance, short range wireless attack and remote wireless attack, and proved the feasibility of these attack methods through experimental demonstration^7^. Liu et al. proved that the systems in the vehicle network (airbag, instrument panel and tire pressure detection system, etc.) were vulnerable to malicious attacks by analyzing some attack behaviors of the vehicle network^8^. Koscher et al. used external network devices such as Bluetooth and WIFI to carry out malicious attacks on the car and control the operating status of the vehicle^9^. Hoppe et al. proposed an attack method that could control the window lift and ABS and other functional systems in automobile CAN bus network^10^. Woo et al. connected the vehicle environment through malicious applications of smart phones to achieve remote wireless attacks to the vehicle network^11^. With the development of intelligent networked vehicles, the security vulnerabilities of automobile CAN bus itself make the security problems of VMNs more serious.

In view of the security problem of the VMN, researchers have also carried out a large number of studies, which can be divided into the following three types. (1) Encryption and authentication based technologies. Wang et al. proposed an authentication framework based on the use of encryption keys to verify the validity of messages^12^. Szilagyi et al. proposed a digital signature based on pairwise symmetric keys to improve the security of CAN protocol^13^. Although encryption and authentication methods can effectively improve the security of onboard CAN bus network, they are often restricted by onboard network computing capacity, bandwidth and storage resources in practical applications^14^. (2) Isolating car interface from the VMN. Macher et al. used the signal interface method to identify the attack vector of automobile system, so as to prevent the malicious attack from accessing and invading the system^15^ . Although such methods can reduce security risks, they are not effective in preventing internal attacks, and it is increasingly difficult to completely isolate intelligent connected vehicles due to the increasing number of external interfaces. (3) Intrusion detection based protection system. Intrusion detection system (IDS) is a reactive countermeasure to identify the network attack. Although the computing and bandwidth resources of VMN are limited, IDS is still a real-time effective and backward compatible security protection measure for the VMN. Choi et al. proposed a vehicle identification system that can identify the message source on CAN bus by detecting the corresponding communication characteristics of CAN signal^16^. Katragadda et al. used CAN identifier to carry out sequence mining so as to detect low-speed injection attack in CAN bus^17^. Taylor et al. proposed a frequency-based anomaly detection method that will send an anomaly alarm signal when the frequency exceeds a preset threshold^18^. Shin et al. successfully detected the DOS attack by using continuous error frame detection method^19^. Suda et al. proposed an IDS based on time series features by taking advantage of the periodicity of CAN bus messages and combining it with the recursive neural network^20^. Muter et al. proposed an entropy-based intrusion detection method to protect the VMNs^21^. Wu et al. proposed a sliding window mechanism of information entropy based on fixed message number in order to understand the problem of non-periodic CAN message entropy^22^. With the development of machine learning, IDS based on machine learning has been favored by more and more researchers. Theissler et al. proposed a single class support vector machine intrusion detection method based on radial basis function kernel^23^. Kang et al. proposed an intrusion detection method based on deep neural network to classify and identify normal and abnormal messages^24^. Khan et al. proposed a VMN intrusion detection method based on long and short-term memory (LSTM) neural networks^25^.

Convolutional neural networks (CNN) were also widely used in image recognition and network anomaly detection. For example, Lopez-Martin et al. proposed a novel randomized CNN model for the detection of the early stages of Alzheimer's disease based on the MEG activity. Experimental results showed that this method obtained the best performance compared to some other classical machine learning algorithms^26^. Lopez-martin et al. proposed an Internet of Things traffic classifier combining CNN and recurrent neural network, which achieved the best detection results compared to other algorithms^27^. Song et al. proposed an IDS based on the Inception-ResNet CNN model, which extracted the CAN ID features in each CAN message as a 29-bit binary then consecutively put 29 of them to form a 29*29 grid data as the input of the model^28^. However, as CNNs are good only in processing image-like grid data, this kind of sequential coding method is not very effective in making full use of the feature extraction capability of the CNN and cannot guarantee the time connection of the CAN ID sequential data. On the other hand, the intrusion detection methods proposed by now were only able to detect one kind of attack. When there are multi-attacks invaded into the network, the same number of detection models would be required to be established to detect each of them. In order to overcome the drawback of the current methods, we proposed a Mosaic-coded CNN model to detect multi-attack intrusions with multi-classification within a single model.

The main contributions of this paper are as follows:

1. In view of the shortcomings of the sequential coding method^28^ in the processing of CAN ID data, a Mosaic coding method was proposed to convert the one-dimensional CAN ID to the two-dimensional grid data for the CNN to effectively extract data features and ensure the time connections of time series data.
2. Instead of detecting one type of attack, datasets containing multiple types of malicious attack were used to train and test the model so that it is able to detect any combination of attacks and to clearly classify each of them.
3. The autoencoder was used to reduce the dimensionality of data and cut down the number of parameters and computational cost of the model to meet the limited computing capacity and storage resources of vehicle-mounted devices.

## Background knowledge

### In-vehicle network

Due to the relatively simple internal structure of early vehicles, the ECU inside the vehicle could only be connected by wires and plug-ins. However, with the development of automobile intelligence, the functions of vehicle system become more and more complex and the number of ECU modules inside the vehicle also increases. If ECUs are directly connected through wires, the number of wires inside the vehicle will increase sharply, resulting in more and more complex wiring, which will cause inconvenience for the fault location and maintenance of the vehicle in the later period and reduce the reliability of it. Therefore, researchers have developed in-vehicle networks based on network structures such as CAN, LIN, FlexRay and MOST, which greatly improve the communication rate and transmission reliability of data inside the vehicle.

**Figure 2:**
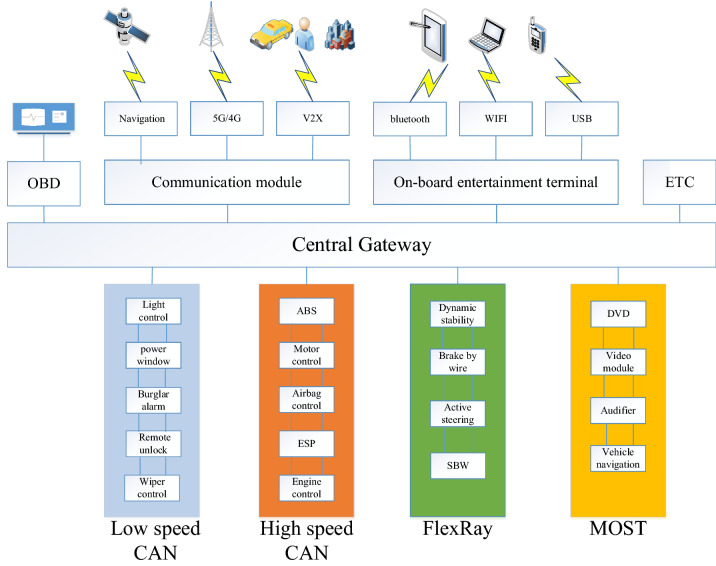
Internet of vehicles network topology.

The electronic devices inside the vehicle are mainly composed of ECUs and actuators. As different systems have different requirements for the communication rate, different ECUs are connected to the data buses with different transmission rate. According to transmission rates, vehicle internal network system can be divided into low speed CAN bus, high speed CAN bus, FlexRay and MOST networks. Gateway is used to connect and communicate between different data buses, thus forming a complex and multi-level network topology inside the vehicle. In the modern Internet of vehicles system, the VMN gradually moves from closed to open, greatly enhancing the information interaction with the network outside the vehicle. Figure 2 shows the network structure of modern vehicles.

**Figure 3:**
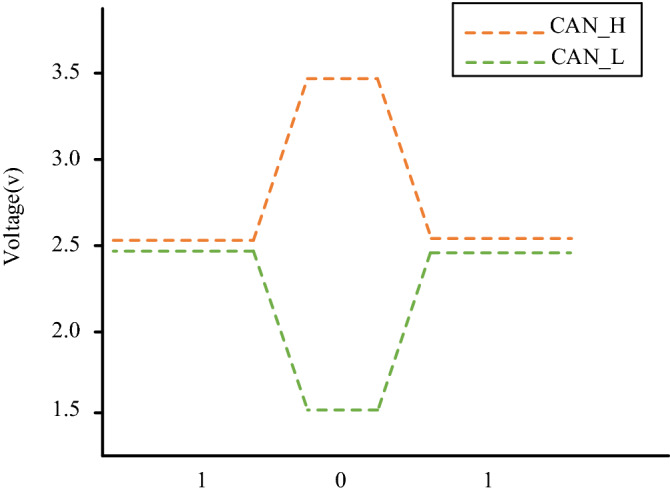
Signal logic of CAN bus, where the orange dotted line represents the high level bus and green dotted line represents the low level bus.

Controller Area Network is a serial data communication protocol developed by German Bosch company from 1980s to solve the internal communication of modern cars, and the CAN2.0 standard was introduced in 1991. CAN bus has been widely used in the field of industrial control for its flexibility, reliability and real-time performance. At present, CAN bus network is the most widely used network protocol in VMN, and has become the de facto standard of the VMN. All nodes on the CAN bus are connected by twisted pairs of high level CAN_H and low level CAN_L. CAN bus protocol adopts differential signal and uses non-zero bit coding mode to encode, where 0 represents dominant bit and 1 represents recessive bit. The signal logic of CAN bus is shown in Fig. 3. When an ECU sends a recessive bit, the voltage of CAN_H and CAN_L is both 2.5 V, and the difference between them is 0 V. When an ECU sends a dominant bit, the voltage of CAN_H is 3.5 V while that of CAN_L is 1.5 V, and the difference between them is 2.0 V.

**Figure 4:**
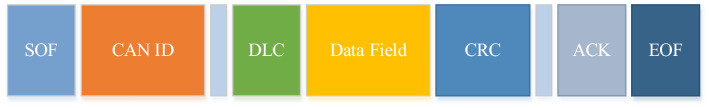
CAN data frame structure, where SOF represents Start of Frame, DLC represents Data Length Code, CRC represents Cyclic Redundancy Check, ACK represents Acknowledgement, and EOF represents End of Frame.

CAN bus protocol is a message-based broadcast protocol. Each CAN message has a CAN identifier (ID). The ECU determines whether to accept the message according to the CAN ID of the message on the bus. When multiple ECU nodes on the CAN bus send messages at the same time and conflict occurs, the CAN bus performs bit-by-bit discrimination on the CAN ID to determine message sending priority according to the principle that the ECU with a smaller value of CAN ID has a higher priority to send messages. To maintain system’s consistency, the ECU periodically broadcasts messages even if data values have not changed, which makes the ECU’s message periodic. CAN bus messages can be divided into four types: data frame, remote frame, error frame and overload frame. The data and remote frames are used to send or request data. The error and overload frames are determined by the established protocol and the actual situation of the network. As data frame is used to transmit data and carries the information of vehicle running condition and the related control information, researchers can use it to detect intrusions to the network. CAN message data frames are divided into two formats: standard frame with 11-bit CAN ID and extended frame with 29-bit CAN ID. Figure 4 shows the structure diagram of the CAN message frame.

### CNN

Convolutional neural network, as a feedforward artificial neural network, is one of the representative algorithms of deep learning, which is widely used in image and audio recognition. CNNs can effectively improve the learning and expression abilities of neural networks by taking advantage of local receptive fields, weight sharing and pooling. The network structure of CNN mainly includes convolution layer, pooling layer and full connection layer.

The convolutional layer achieves feature extraction of input data through the convolution kernel, and the neural units in the convolution kernel are sparsely connected. The parameters of convolution layer are updated by back propagation algorithm. The convolution process is to find the sum of the weight of the convolution kernel and its corresponding element on the input feature map using the following formula:

1 $$conv_{x,y} = \mathop \sum \limits_{i}^{{p{*}q}} w_{i} v_{i}$$

where (x, y) are the image space coordinates, p*q is the convolution kernel size, w is the weight of the convolution kernel, and v is the image’s brightness value.

The size of the output feature graph of the convolution layer is determined by the hyperparameters of the convolution kernel size, step size and filling method. The size of the convolution kernel can be set to any value smaller than the size of the input feature map. The larger the convolution kernel is, the more complex the input features being extracted are. The convolution step defines the distance between the two adjacent feature images scanned by the convolution kernel. When the step is n, the convolution kernel will skip n-1 pixels in the next scan. The purpose of filling is to offset the impact of image size reduction. The common filling method is to use 0 to fill the image’s boundary.

After the convolution operation, a bias b is usually added and a nonlinear activation function $h\left( x \right)$, is introduced. The calculation formula of feature activation function is as follows:

2 $$z_{x,y} = h\left( {\mathop \sum \limits_{i}^{{p{*}q}} w_{i} v_{i} + b} \right)$$

Pooling is a kind of nonlinear down-sampling operation. After the input data is extracted by the convolution layer, in order to avoid the problem of low model efficiency and easy overfitting caused by the excessively high data feature dimension, dimension reduction of data is carried out by using the pooling operation. The reduction of sampling can be divided into maximum pooling and average pooling. The former simply takes the maximum of a subset of the input as the output but the latter takes the mean of it.

The full connection layer of CNN is equivalent to the traditional feedforward neural network. After the pooling layer, the feature graph is flattened into a vector to implement the full connection. In CNN, the function of this layer is to perform a nonlinear operation of the extracted features to obtain the output. That is, the full connection layer itself is not expected to have the feature extraction ability, but attempts to use the existing high-order features to achieve the learning goal.

### Intrusion detect system

**Figure 5:**
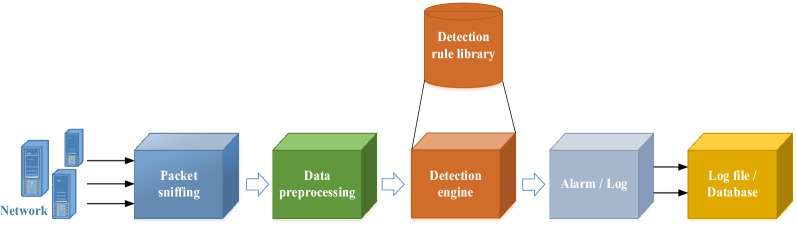
Flow chart of the IDS.

IDS is a common network security protection measure. It can monitor network’s transmission in real time, and when the system finds abnormal conditions, it will send out an alarm or take active response measures. According to the intrusion detection behavior, IDS is divided into anomaly detection and misuse detection. Anomaly detection uses a normal database to compare the behavior data generated in the network and detect abnormal attacks. Misuse detection uses an abnormal behavior database that contains all the known attacks to match the input data and detect attacks. These two detection strategies have their own advantages and disadvantages. The failure rate of anomaly detection is low, but the false positive rate is high because all the data that does not meet the database behavior pattern are judged as attacks. Misuse detection directly compares network behavior data with abnormal attack database, so the false positive rate is low. However, if the abnormal behavior database is not updated in real time, the failure rate will be high. The workflow of the IDS is shown in Fig. 5.

## Method

### Two-dimensional Mosaic pattern based encoding

**Figure 6:**
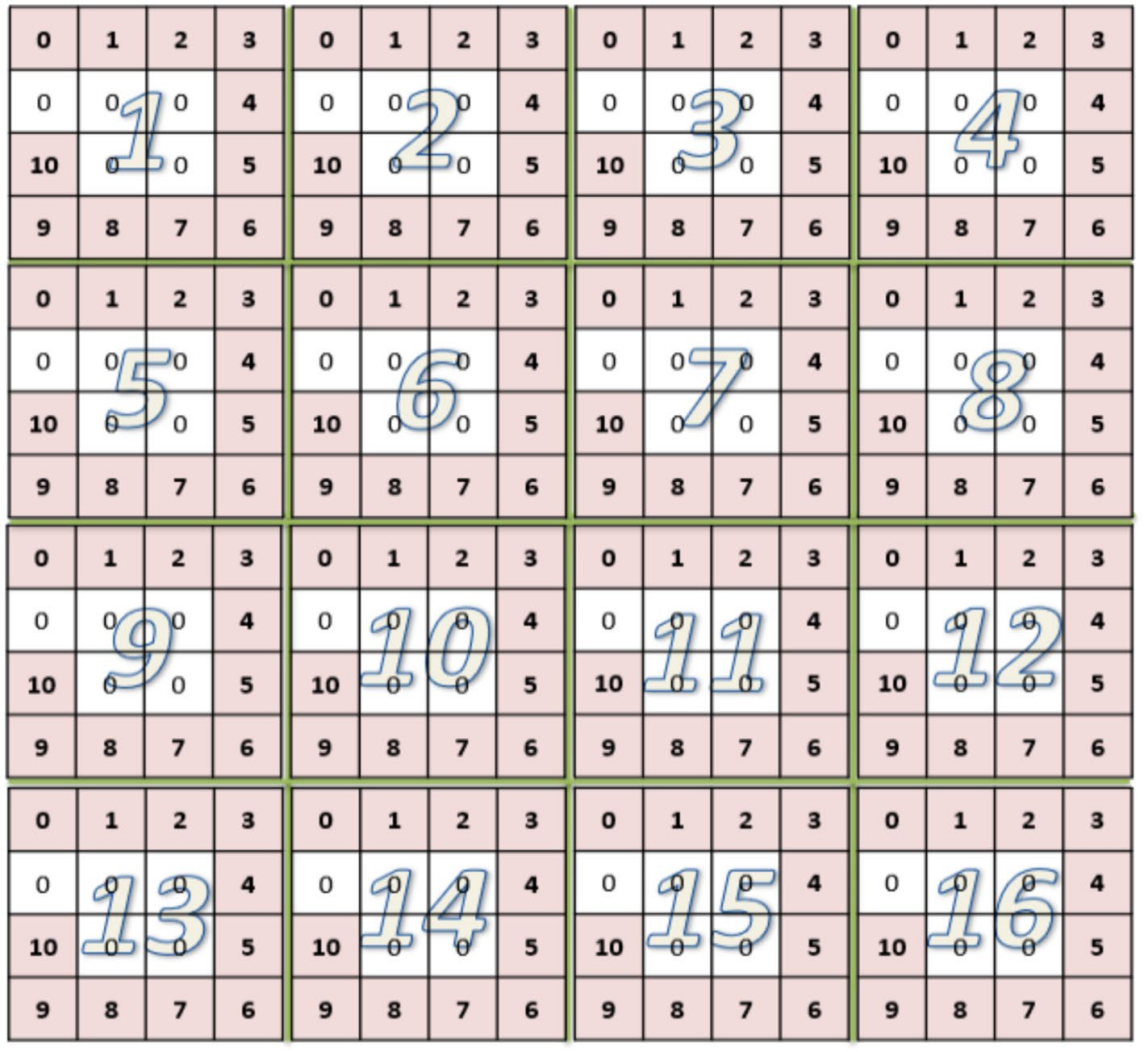
16 4*4 data grids arranged sequentially one after another to form a Mosaic data structure as the network input to keep the time feature of the original data.

In order for the CNN to successfully process the data, each one-dimensional binary CAN ID was first converted to a 2D data grid then a number of such data grids were put together to form a two-dimensional Mosaic pattern. For a 11-bit CAN ID, a 4*4 data grid was formed in which the 11-bit binary numbers were first filled around the 4 edges of the grid and then the remaining 5 elements were filled by zero. The reason of filling the data in this way is that the patterns surrounding the grid could more easily be recognized by the CNN. The two-dimensional data grid was then further arranged to form a larger two-dimensional Mosaic structure in the form of 8*8 data patterns, a reduced version of which is shown in Fig. 6 where the numbers in the grid represent the bits in binary. In this Mosaic data structure, the temporal relationship among sequential data features was kept by arranging one data grid after another from the continuous data series. 64 such 4*4 data grids form a 32*32 element Mosaic pattern. The converted Mosaic data patterns will be used as the training and test sets of the CNN to preserve the time characteristics of the original data.

**Figure 7:**
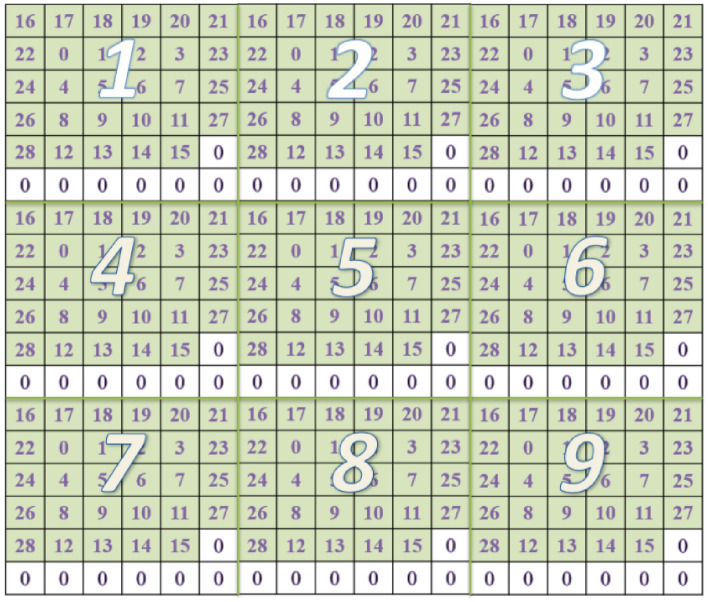
Results of 3*3 Mosaic pattern data arrangement with a 29-bit CAN ID coded in a 6*6 grid.

If the 29-bit CAN ID data was used, we would convert each piece of data to a 6*6 data grid. Currently, as only the first 11 bits of data in the total 29 bits would be nonzero, we put them around the center of the 6*6 grid to make the most of CNN’s capability of recognizing 2D patterns. Since there are 36 elements in the 6*6 grid, 29 of them were filled by the CAN ID and the remaining 7 elements were filled with zero. Similarly, after the data were gridded, they were put together to form a Mosaic structure to maintain the time characteristics between each piece of data. In this paper, 8*8 data grids were used to form a 48*48 Mosaic structure, which served as the input of the training and test sets of the network. Since the 8*8 data grids is too large, Fig. 7 only shows the result of 3*3 data grids to form an 18*18 Mosaic structure, where the numbers in the grid also represent the bits in binary.

### CNN model

The CNN model used in this paper consists of an input layer, a convolution layer, a pooling layer, a full connection layer and an output layer. The input is the two-dimensional Mosaic grid data described in the previous two subsections.

In the convolution layer, 20 convolution kernels were used for operation. The output size of the convolution layer can be worked out using Eq. (3), where O is the output size, W is the data size, K is the kernel size, P is the padding method, and S is the step size. The padding P was used to ensure that the data size after convolution is the same as that of the input. The dimension of pooling window F was 2*2, and average pooling was used to take the average value of data in each non-repeated 2*2 region. Therefore, the dimensionality of output data after pooling was reduced to half of the original one.

3 $${\text{O}} = \frac{W - K + 2P}{S} + 1$$

The input of convolution is depended on the output of the last layer and the output of it determines the input of next layer. When there are multiple convolution layers, the output of each convolution layer can be worked out by Eq. (4), where $a^{i}$ is the output of a convolution layer, $\sigma$ is the activation function, $a^{i - 1}$ is the output of the last layer, $W^{i}$ is the connection weight, and $b^{i}$ is the bias of the layer. In this paper, the bias $b^{i}$ was set with an initial value of 0.1.

4 $$a^{i} = \sigma \left( z \right) = \sigma \left( {a^{i - 1} {*}W^{i} + b^{i} } \right)$$

Then there is the full connection layer. 128 neurons were used in the full connection layer which were connected to the neurons of the flattened final pooling layer. The bias value b in the 128 neurons was also set with an initial value of 0.1, and tanh was used as the activation function. In order to prevent over-fitting in the process of model training, a dropout layer was used which was realized by making the activation function in some neurons fail with a certain probability. In training process, the failure probability in the dropout layer was set as 0.5, but during the test, it was equal to 0, i.e., no dropout at all in the test stage.

Finally, it is the output layer. Because we have five types of data including normal and DOS, Fuzzy, RPM and Gear attacks and our objective is to accurately classify each of them, the output layer also contains five neurons. The softmax classifier shown in Eq. (5) was used at this layer to classify these five categories, as it is more suitable for multiple classification tasks than other activation functions.

5 $$P_{i} = \frac{{e^{{o_{i} }} }}{{\mathop \sum \nolimits_{i = 1}^{n} e^{{o_{i} }} }}$$

where $n$ is the number of classes ($n = 5$), $o_{{i{ }}}$ is the output of neuron $i$ and $P_{i}$ is the probability of neuron $i$.

**Figure 8:**
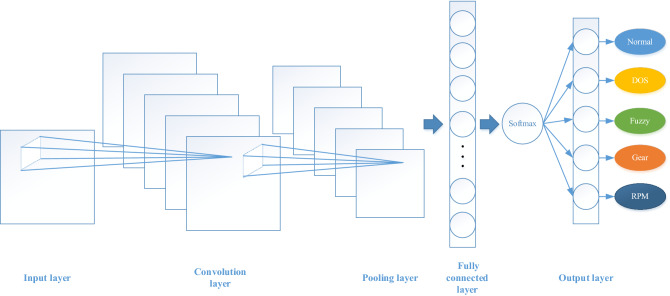
The CNN model structure used in this paper.

One-Hot encoding was used that takes the largest output neuron as the type of the model output. The structure of CNN is shown in Fig. 8. In the optimization of the CNN, the cross entropy loss function was selected and the adaptive moment estimation optimization method was used to minimize the function. By constantly adjusting the learning rate, the optimal learning rate of the optimizer was found to be 1e-4.

The model was trained and tested using the VMN attack data collected by Hacking and Countermeasure Research Lab in South Korea^29^. This data includes four datasets: DOS, Fuzzy, Spoofing Gear and Spoofing RPM attacks. The data recorded CAN traffic through the OBD-II port of a real vehicle when the attack messages were injected. During the process of data collection, the engine of the test vehicle remained on. Each dataset had a total of 30–40 min of CAN traffic and contained 300 intrusions of the injected message, each continued for 3–5 s.

The proposed CNN model was tested in a series of experiments. In order to evaluate the effectiveness of the proposed models, a combination of the four datasets mentioned above was used as the training and test sets to meet the requirements of multi-type attack detection. During the Mosaic coding, in order to ensure the temporal connectivity between the CAN ID sequence, we arranged the grid of 3*3, 4*4 or 6*6 CAN IDs in chronological order to form a Mosaic pattern. After all CAN ID data have been encoded in the Mosaic form, the model randomly selects a Mosaic block during the training and test process in order not to be affected by the data arrangement. The training set contained a randomly selected 75% of the data and the remaining 25% was used as the test set. Although the model was trained using the combination of all four sets of data, in test phase, all the possible combinations of different types of datasets were used for test. Therefore, the test sets contained $C_{4}^{1}$ = 4 combinations of one kind of attack, $C_{4}^{2}$ = 6 combinations of two kinds of attacks, $C_{4}^{3}$ = 4 combinations of three kinds of attacks, and $C_{4}^{4}$ = 1 combination of four kinds of attacks, with a total of 15 different attack types. Each dataset and its index number are shown in Table 1. With this type of training and test, our model would be able to detect any intrusion combinations in the future, which is different from the current ones which were only able to detect a single type of attack after being trained.

**Table 1:** Dataset combinations and their numbers.

Number	DataSet	Number	DataSet
*1*	*Normal_DoS_Fuzzy_Gear_RPM*	*9*	*Normal_Fuzzy_Gear*
*2*	*Normal_DoS_Fuzzy_Gear*	*10*	*Normal_Fuzzy_RPM*
*3*	*Normal_DoS_Fuzzy_RPM*	*11*	*Normal_Gear_RPM*
*4*	*Normal_DoS_Gear_RPM*	*12*	*Normal_DoS*
*5*	*Normal_Fuzzy_Gear_RPM*	*13*	*Normal_Fuzzy*
*6*	*Normal_DoS_Fuzzy*	*14*	*Normal_Gear*
*7*	*Normal_DoS_Gear*	*15*	*Normal_RPM*
*8*	*Normal_DoS_RPM*		

### Autoencoder

Due to the limitation of computing and storage capacity of the vehicle equipment, it is better to reduce the model complexity as far as possible. Therefore, autoencoder network was used to reduce the dimension of data, so as to effectively cut down the number of model parameters as well as the computational complexity. Autoencoder is a kind of unsupervised neural network and a common data dimension reduction method. It consists of an encoder and a decoder. The encoder compresses the input data into a low-dimensional space, which is then restored by the decoder to the original data. The encoder and decoder are connected to form a neural network, and the network parameters are optimized by back propagation algorithm. If we denote the encoder as $\emptyset$ and the decoder as φ, the structure of the network will take the following form:

$$\emptyset :X \to Z$$

$$\varphi :Z \to X$$

6 $$\emptyset ,\varphi = arg\mathop {\min }\limits_{\emptyset ,\varphi } \, || X - \left( {\emptyset { }^\circ \varphi } \right)X  || ^{2}$$

**Figure 9:**
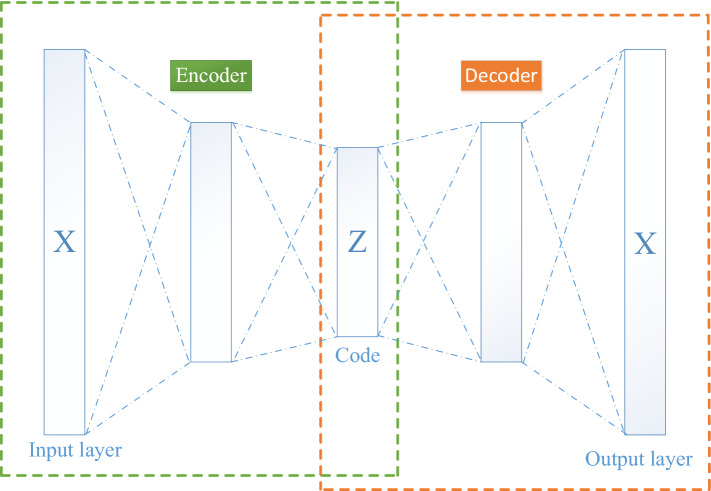
Network structure of the autoencoder used in this paper.

The network structure of the autoencoder used in this paper is shown in Fig. 9. The input X is the 29-bit CAN ID data that will be compressed to the 9bit feature data by the autoencoder. Then the 9bit data will be converted to the 3*3 grids and 8*8 of such data grids will be used to form a 24*24 element Mosaic pattern, which will be used as the input of the CNN model. The number of hidden neurons in our autoencoder was 17. During the training process, the output $X_{Pred}$ and input $X_{True}$ were used for error calculation and the mean-square error was adopted as the error function. The formula is shown in Eq. (7). The model adopted Adam optimizer and the learning rate was set to 0.001 for parameter optimization. Moreover, the small batch gradient descent method was adopted with a batch size of 200 for error calculation, and the model was trained for 5 epochs. After the model training was completed, the intermediate feature data Z was extracted and saved to a local file as the input of the CNN.

7 $$MSE = \frac{1}{n}\mathop \sum \limits_{i = 1}^{n} \left( {X_{Pred} - X_{True} } \right)^{2}$$

## Experimental results and discussions

### Experimental results

Using the CNN models, we tested both the 11-bit and the 29-bit CAN IDs. No matter whether the 11-bit or 29-bit CAN ID was used, the CAN IDs were encoded by both the sequential coding method described in^28^ and our Mosaic coding method described in the previous section.

**Figure 10:**
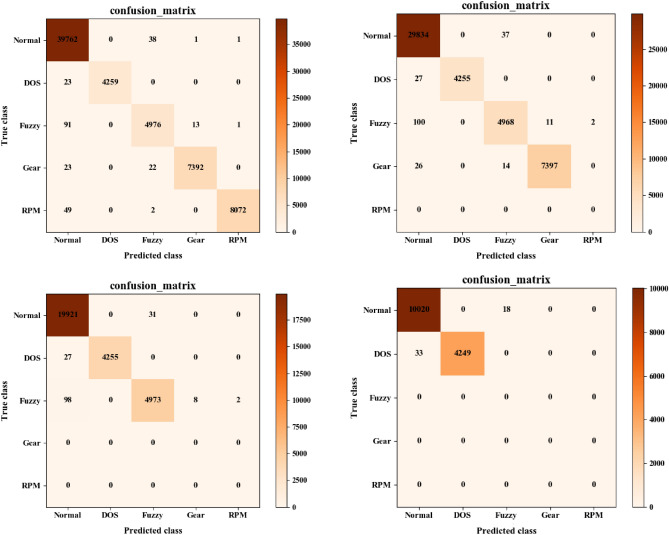
Results of the typical confusion matrices using Mosaic coding for 11-bit CAN ID. The numbers in the confusion matrix represent the number of samples from the original category represented by the horizontal axis being classified into the category represented by the vertical axis. For example, the intersection between Fuzzy (vertical) and Normal (horizontal) represents the number of Normal samples wrongly classified by the model as Fuzzy.

**Figure 11:**
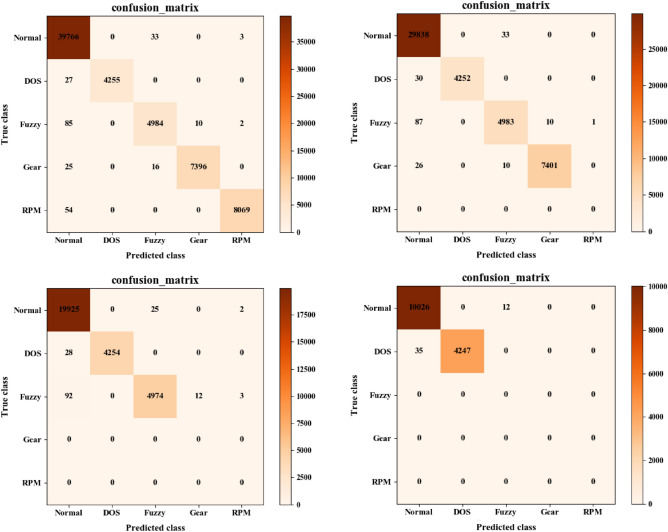
Results of the typical confusion matrices using Mosaic coding for 29-bit CAN ID. The numbers in the confusion matrix represent the number of samples from the original category represented by the horizontal axis being classified into the category represented by the vertical axis. For example, the intersection between Fuzzy (vertical) and Normal (horizontal) represents the number of Normal samples wrongly classified by the model as Fuzzy.

Figures 10 and 11 represent some typical confusion matrices results by Mosaic coding method for 11-bit and 29-bit CAN IDs on different attack data combinations respectively. From left to right, there are four attack types (DOS, Fuzzy, Gear, RPM), three attack types (DOS, Fuzzy, Gear), two attack types (DOS, Fuzzy) and one attack type (DOS) respectively. In these figures, the digit in each cell represents the number in a horizontal type (predicted type) being predicted to the vertical type (True type). For example, digits in the first column of the confusion matrix represent the numbers of different types being predicted to RPM attack by the model, and the digits in the diagonal represent the correctly classified number by the model. It can be intuitively seen from these figures that the trained model in most of the cases could correctly detect different attack types. However, when the dataset contained multiple attack types, an attack type would sometimes be misclassified as other types and it was the easiest for the model to misclassify an attack type as Normal. On the other hand, when the dataset did not contain any attack, the model may misclassify it as an attack. These misclassifications would slightly reduce the model’s prediction accuracy.

In order to more precisely evaluate the model’s prediction performance, evaluation indexes similar to the binary classification model, i.e., Accuracy, Recall, Precision and F1_score were also worked out according to the confusion matrix for each category. In the multi-classification model, a certain category (such as Normal) is defined as a positive class and the remaining categories (DOS, Fuzzy, Gear and RPM) are all defined as negative (Intrusion) classes, and different positive and negative classes are selected successively. Then, the True Positive (TP), False positive (FP), False negative (FN), and True negative (TN) can be worked out for each type of attack. The relevant evaluation indexes are calculated according to the formulas of the corresponding evaluation indexes in dichotomies, which are given in (8)—(11), where *E* represents the set of attacks tested in a specific case.

8 $${\text{Accuracy }}\left( {{\text{Acc}}} \right):\;\;\;\;\;\;\;\;\;\;\;\;\;\;\;{\text{Acc}} = \frac{{\mathop \sum \nolimits_{i \in E} {\text{TP}}_{i} }}{{{\text{SUM}}}}$$

9 $${\text{Recall }}\left( {{\text{Rec}}} \right):\;\;\;\;\;\;\;\;\;\;\;\;{\text{Rec}} = \frac{{\mathop \sum \nolimits_{i \in E} {\text{TP}}_{i} }}{{\mathop \sum \nolimits_{i \in E} \left( {{\text{TP}}_{i} + {\text{FN}}_{i} } \right)}}$$

10 $${\text{Precision }}\left( {{\text{Prec}}} \right):\;\;\;\;\;\;\;\;\;\;{\text{Prec}} = \frac{{\mathop \sum \nolimits_{i \in E} {\text{TP}}_{i} }}{{\mathop \sum \nolimits_{i \in E} \left( {{\text{TP}}_{i} + {\text{FP}}_{i} } \right)}}$$

11 $${\text{F1}}\_{\text{score}}:\;\;\;\;\;\;\;\;\;\;\;{\text{F}}1\_{\text{score}} = \frac{{2{\text{*Prec*Rec}}}}{{{\text{Prec}} + {\text{Rec}}}}$$

**Figure 12:**
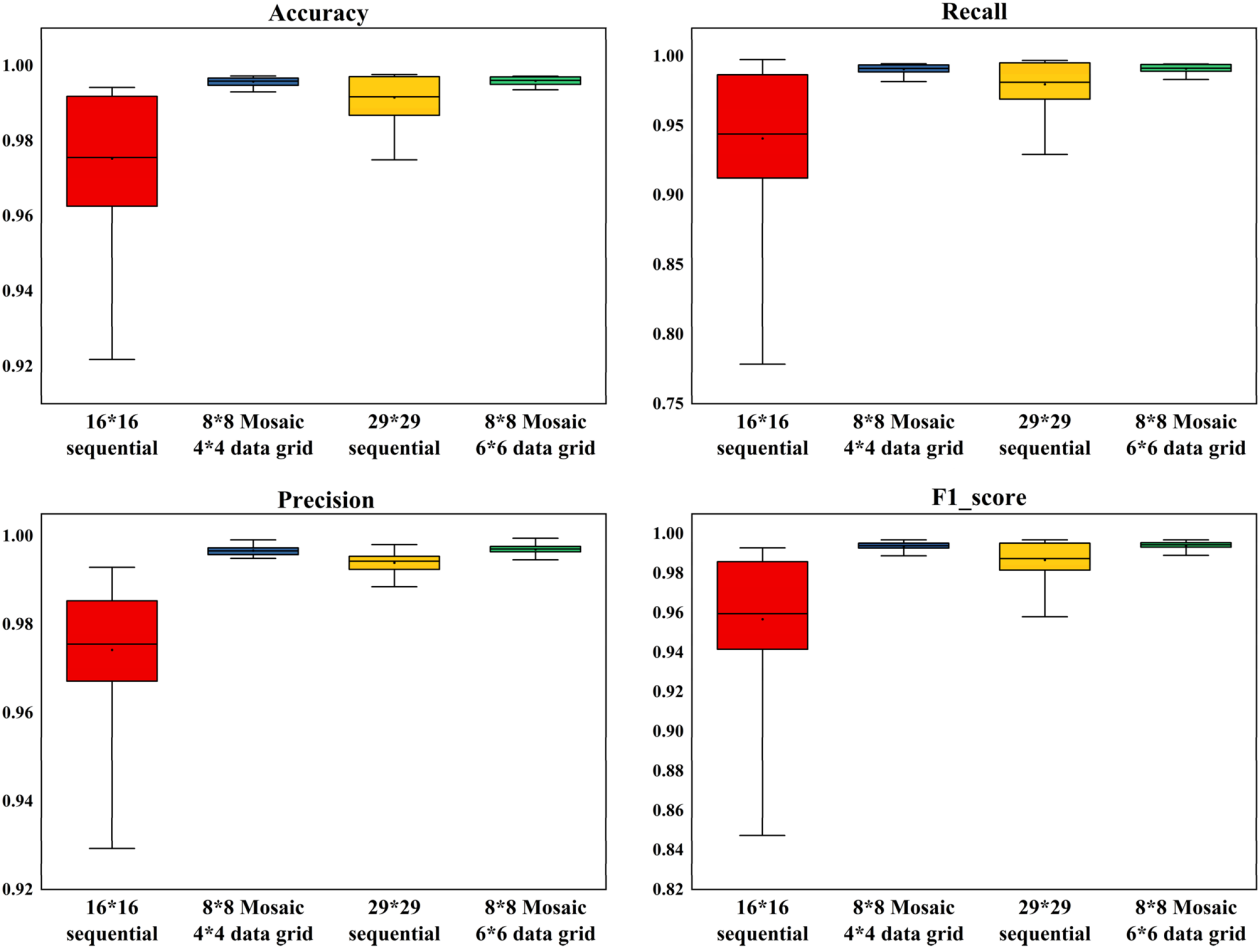
Comparison of box diagram for the test results of various indicators in different methods with the 15 different invasion data combinations given in Table 1.

Based on the above confusion matrices, formulas (8)-(11) can be used to calculate the evaluation indexes of the model in datasets containing different attack combinations. Figure 12 gives the box diagram of overall classification Accuracy, Recall, Precision and F1_score tested by different coding methods with all the data combinations given in Table 1. We can see from this figure that, no matter whether the 11-bit or 29-bit CAN ID was used, our Mosaic coding methods is superior to sequential coding method with much lower variance in all evaluation indexes, which showed that the Mosaic coding method proposed in this paper has better classification ability when facing multiple types of attacks. When the 11-bit CAN ID was used, the performance obtained from the sequential coding method fluctuated greatly for different kinds of invasion combinations, while the test results obtained from the Mosaic coding method were very stable with higher performance no matter what kind of invasion combination was used. When the 29-bit CAN ID was used, although such fluctuation obtained from the sequential coding method was gentler, the results obtained from our method were equally better with stable high performance.

**Figure 13:**
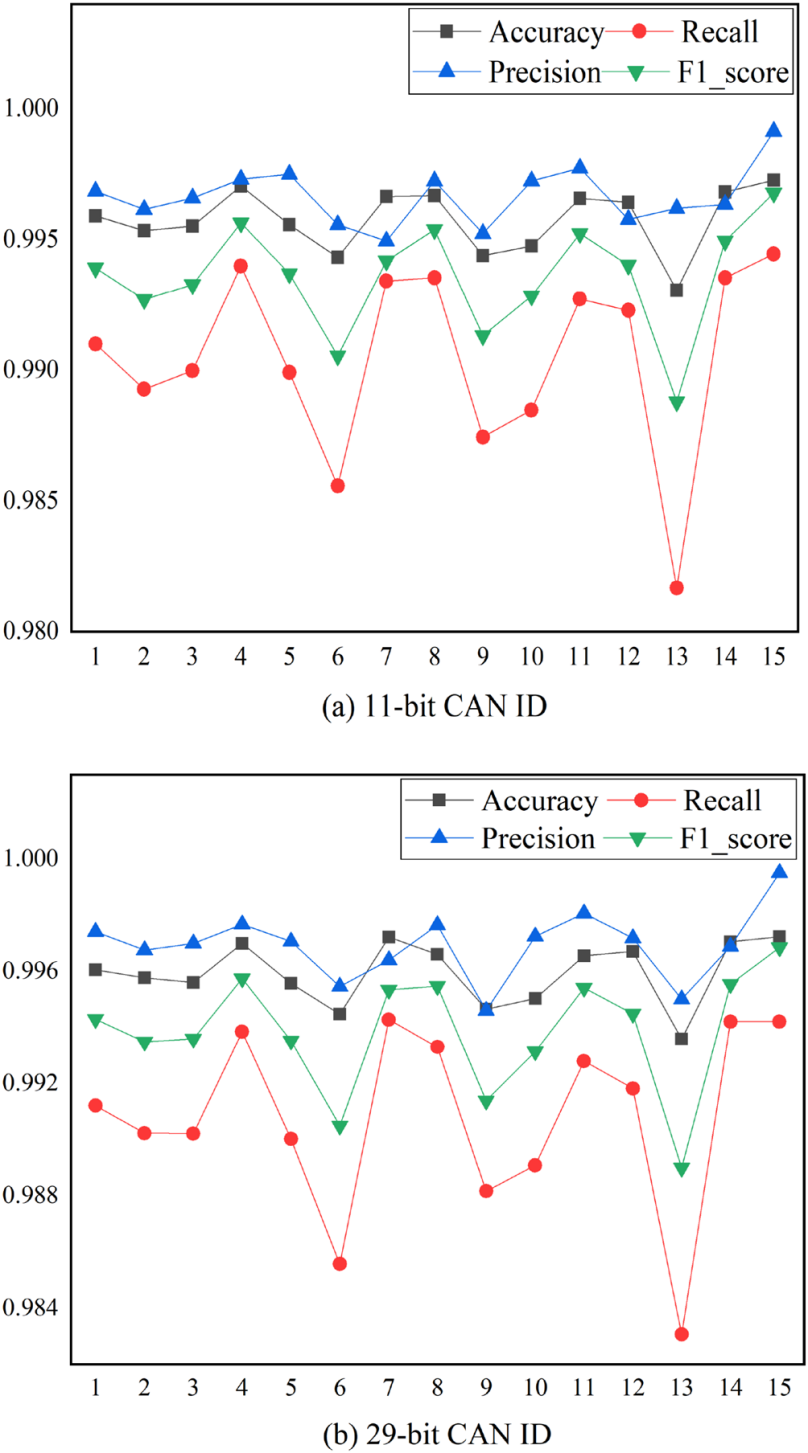
Test results of Mosaic coding methods under different invasion combinations.

Figures 13a,b shows the model test results of Mosaic coding method under different attack combinations. The abscissa numbers in the figure correspond to each combination attack dataset in Table 1. As can be seen from the figure, regardless of 11-bit or 29-bit CAN IDs, the Mosaic coding method has good performance under different attack combinations. When the dataset contains Fuzzy attack, the performance would decline. The portion of Fuzzy attack in the dataset gradually increased from set 1 to 2, 3, 5 to 6, 9, 10 and to 13, the performance of the model also decreased in the same order, which showed that the Fuzzy attack is the most difficult to be detected.

**Figure 14:**
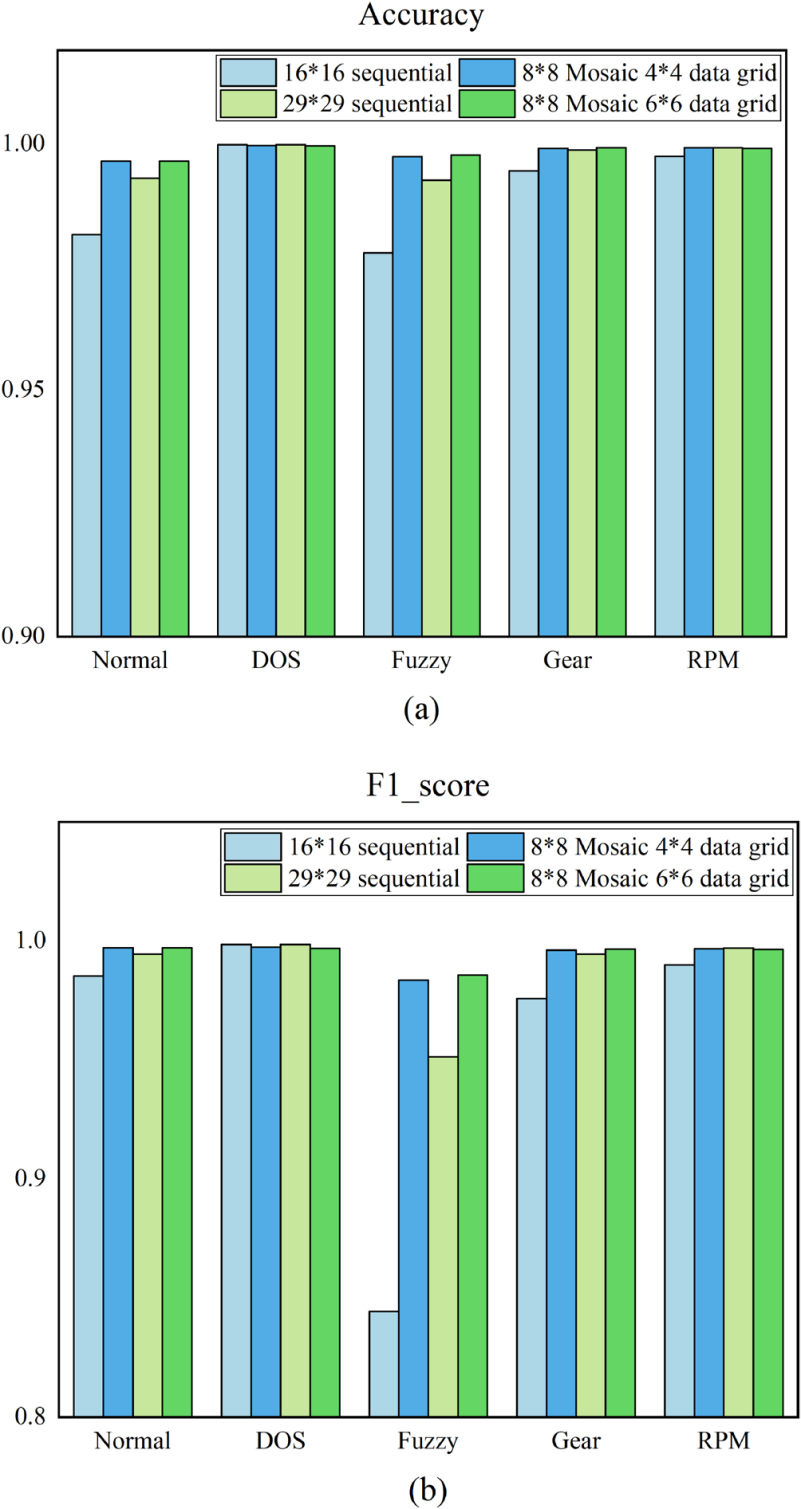
Results of each attack type using different coding methods on dataset no. 1.

Figures 14a,b shows the evaluation index results of each attack in dataset number 1 with different kinds of coding methods. As can be seen from the figure, the experimental results of Mosaic coding method are generally better than the sequential coding, especially for Fuzzy attack, the accuracy and F1_score in Mosaic coding were greatly improved. But there are also some cases where the direct sequential coding method performed slightly better than the Mosaic coding method. This is because the results from the sequential coding method were good enough in these cases, which were difficult to be further improved by the Mosaic coding method. This showed that in most of the cases, the proposed method can more effectively identify any attack type contained in the dataset.

## Discussions

**Figure 15:**
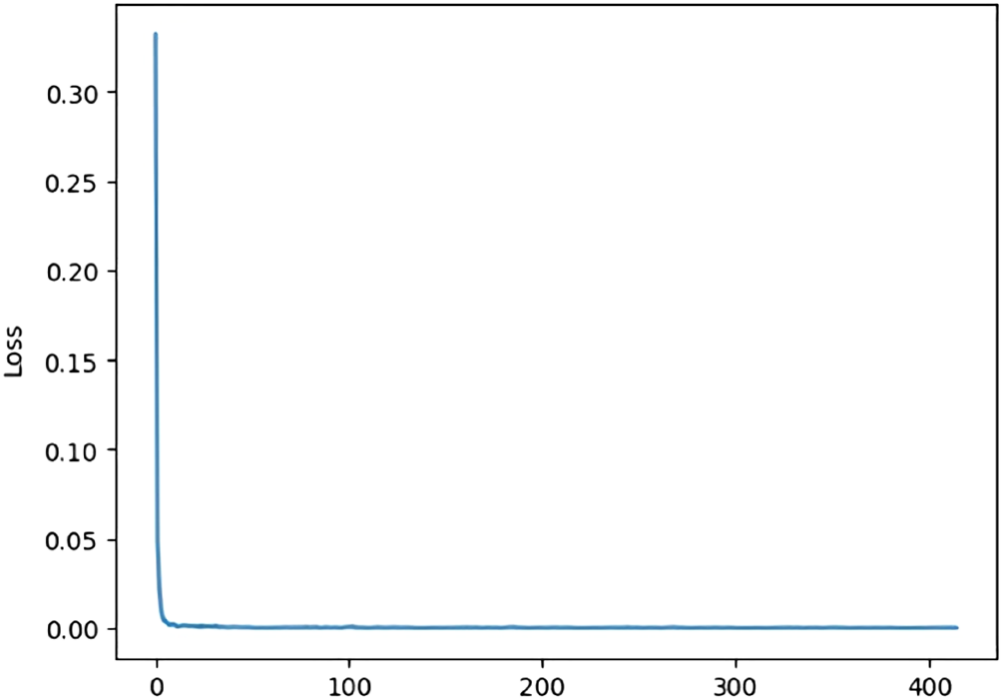
Change of autoencoder’s loss after five epochs of training.

As CAN ID contains a large number of zeros, especially for the 29-bit CAN ID, when they are directly used to create grid data for model training, there would require a large number of model parameters and consume large quantity of computational resources. Therefore, autoencoder was used to extract features of data, and then the features were used to train the CNN model for classification and discrimination. Figure 15 shows the change of autoencoder’s loss after five epochs of training. We can see from this figure that the autoencoder converged quickly after a few epochs of training.

**Figure 16:**
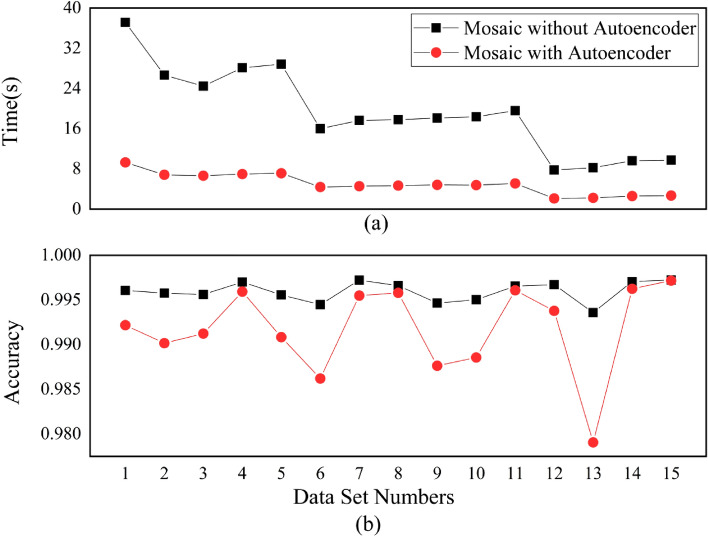
Comparison of model’s test running time and the overall accuracy under different invasion combinations with and without autoencoder for dimension reduction.

After the autoencoder had been trained, the 29-bit CAN ID was compressed to a feature of 9 bits, and then the 9-bit data was used to create a 3*3 data grid with 8*8 such grids being used to form the Mosaic pattern for the CNN training. The trained CNN was used to test the data and obtain relevant evaluation results. The results were compared with those of the 8*8 Mosaic 3*3 data grid without autoencoder feature extraction, so as to evaluate the performance of autoencoder on the model’s detection capability. Figures 16a,b showed the comparison of test running time and the overall model accuracy respectively for the network with and without the autoencoder using the 15 different combinations of datasets with the numbers given in Table 1. As is shown in Fig. 16a, the test running time was greatly influenced by the number of combined attacks for the model without the autoencoder. Dataset 1 contained four types of attacks with the largest number of samples requiring the longest running time; but datasets 12, 13, 14 and 15 contained only one type of attack with the least number of samples requiring the shortest running time. However, when the autoencoder was used, the model test running time was not only greatly reduced but also not influenced much by the number of samples due to much fewer number of connections being used in the model. This suggests that with the reduction of model parameters, the model’s computational complexity was greatly reduced, which would effectively cut down the detecting time and improve the model’s ability for the real-time detection of attacks. Figure 16b shows that, with the reduction of model parameters, the overall accuracy for the network with the autoencoder also slightly declined although it still remained at the acceptable high level. These results showed that the dimensionality reduction by the autoencoder can effectively reduce the computational complexity of the model on the premise that its detection performance is not greatly affected, which is significant when working in the VMN as it only has limited computation power and storage resources.

## Conclusion

Intrusion detection is very important in the safe running of modern intelligent connected vehicles. In view of the deficiency of the existing detecting systems, which can only detect one type of attack, a Mosaic-coded CNN model was proposed to identify and detect intrusions containing multiple attack types. Both the 11-bit and 29-bit CAN IDs were used as the intrusion data and the Mosaic-like two-dimensional data grid was created from them so that the CNN can more effectively extract the data features and maintain the time connections between the CAN IDs. The sequential coding method was also used for comparison purpose. Moreover, dimensionality reduction was carried out on the data using the autoencoder to overcome the limitation of computational resources on the vehicle-mounted equipment and cut down the detection time. Experimental results showed that the Mosaic coding method proposed in this paper can effectively classify and discriminate intrusions containing multi-types of attack combinations with better and more stable performance compared to the sequential coding method.

However, there are also some limitations on the current method. First of all, the dataset adopted in this paper was the integration of those that simulate the situation containing only single attacks. In the future, a dataset that can implement multiple attack types in real time on site need to be adopted or collected for the training and test of the model. Secondly, since only the 11-bit CAN ID was effective in the current dataset, all the simulations that used the 29-bit CAN ID are required to fill the rest number of bits by zero in the coding process. Therefore, some improvements may be made when there is able to collect the real 29-bit CAN ID datasets in the future. By using the real 29-bit CAN ID datasets, we believe that our Mosaic-coded CNN method would perform even better compared to the sequential coding method. Finally, only a relatively simple CNN model was adopted in this paper. If a more complex CNN model structure can be used in the future, our method would be able to identify some more complex intrusions.

Our research showed that although the original data is only one dimensional, the CNN model is still able to extract the required patterns if it is appropriately coded. Experimental results demonstrated that in the detection of malicious attacks to the CAN bus, the Mosaic-coded CAN IDs are better than the sequential-coded ones in achieving a better detection performance. Therefore, apart from using high-performance CNN models, designing a suitable coding method is also important in extracting useful patterns from sequential data.
